# Population Distrust of Drinking Water Safety. Community Outrage Analysis, Prediction and Management

**DOI:** 10.3390/ijerph16061004

**Published:** 2019-03-20

**Authors:** Marco Dettori, Antonio Azara, Erika Loria, Andrea Piana, Maria Dolores Masia, Alessandra Palmieri, Andrea Cossu, Paolo Castiglia

**Affiliations:** 1 Department of Medical, Surgical and Experimental Sciences, University of Sassari, 07100 Sassari, Italy; madettori@uniss.it (M.D.); piana@uniss.it (A.P.); mdmasia@uniss.it (M.D.M.); luca@uniss.it (A.P.); andreacossu@uniss.it (A.C.); castigli@uniss.it (P.C.); 2 Department of Architecture, Design and Urban Planning, University of Sassari, 07100 Sassari, Italy; loria.urbanistica@gmail.com

**Keywords:** drinking water quality, community outrage management, potable water, water for human consumption

## Abstract

The aim of the work is to evaluate the effectiveness of the risk communication strategies in cases of unsafe drinking water supply in Sardinia, the Italian context with the highest population distrust in drinking water safety. During the period 2010–2015, the ordinances published on the institutional websites were analyzed, and the population risk perception was evaluated by applying, for the first time in public health threats, the “OUTRAGE Prediction & Management” software released by Sandman. Overall, 417 ordinances issued by the Sardinian Municipalities were found. Only 1.5% of the ordinances reported information about parameters, concentrations, and risks to health, whereas 4.8% indicated only the parameters and non-standard levels. By contrast, 53.2% specified only the non-standard parameter, and 40.5% indicated a generic non-drinking motivation. The outrage assessment showed values exceeding the threshold of risk acceptance, attributable to the lack and low clarity of the information reported by the ordinances. The present study allowed us to highlight critical issues in risk communication of the quality of drinking water.

## 1. Introduction

Nowadays, water intended for human consumption is still affected by quantitative and qualitative abnormalities. Overall, 2.1 billion people worldwide still have no access to safe drinking water at home, whereas 1.8 billion people drink water with heavy fecal pollution [1,2].

The European Commission has recently proposed a revision of the European drinking water legislation, the European Directive 98/83/EC [3], with the aim of improving the quality of drinking water and the access to it, as well as providing better information to citizens [4]. Increasing communication transparency becomes a key point of the new rules proposed for drinking water, as well as upgrading the quality standards, and protecting the water supply systems, which are known to be critical infrastructures. Accordingly, informing citizens about the quality of tap water should be accompanied by studies on the risks to public health aimed at i) developing methods to be applied in risk analysis; ii) ensuring the safety of the water supply [5,6].

The risk communication process is key in public health, and the European Union is focusing on strategies capable of communicating potential risk in a timely manner, providing messages to be understood by specialist audiences (i.e., policymakers, the scientific community, and industry) but also by consumers in the European Union. Accordingly, recent guidelines published by the European Food Safety Authority (EFSA) [7] focused on the relevant role played by clear, timely and relevant communication strategies. In other words, effective risk communication can contribute to the success of a risk management program by ensuring that consumers are aware of the risks associated with a product and thereby use or consume it safely.

In Italy, Legislative Decrees 31/2001 [8] and 27/2002 [9] implemented the European Directive 98/83/EC and at present represent the reference laws for drinking water. According to the above-mentioned legislative acts, both Europe and Italy have instituted the possibility to request derogation, allowing for the safe management of a systematic exceeding of parameter values, mostly related to geological elements found in water.

For years, as stated by Directive 98/83/EC, the European Union provided derogations to Member Countries whose drinking water quality did not comply with legislative standards. Derogations were aimed at giving time to implement resolutive interventions and restore the drinking water quality. 

As a result, the “derogation arrangement” has been largely used in Italy, with a number of requests higher than any other European Country [10]. Nevertheless, even though most of the problems encountered have been solved, to date, some regions in Italy are still facing difficulties to comply with the legislative standards. There could be many reasons for this phenomenon: (i) the more restrictive parameter values; (ii) the geological contaminants found in many aquifers; (iii) the fragmentation of the aqueduct systems; (iv) the absence of alternative water supplies [11]. 

Thus, once derogations were no longer granted, public notices and municipal ordinances issued to limit the consumption of unsuitable drinking water began to proliferate [10]. Indeed, Italian law states that in cases in which there are hazards for public health, such as the contamination of drinking water, the Health Authority must issue urgent ordinances, which must be temporary measures [12,13,14].

Nonetheless, the contents and format of the ordinances, which are specifically adopted to inform the population about health risks arising from the consumption of unsafe drinking water, are not regulated, and precise laws do not exist. For this reason, the emergence of a growing distrust among citizens towards the consumption of potable water is also attributable to inappropriate strategies of risk communication [15,16]. By contrast, the European Centre for Disease Prevention and Control (ECDC) recently released a literature review [17], based on the study of several theoretical models of risk communication, which underlined that the bridge between academic research and risk communication needs to be strengthened and better communicated to end users and built into risk communication planning and guidance documents (e.g., the ordinances). 

According to Peter Sandman, drinking water may be perceived as an “outrageous” threat [18], dependent on emotional dynamics detailed in his “hazard vs outrage” theory [19]. Thus, the perception of risk is not only a function of “hazard” (magnitude per probability) but it is also closely related to the emotional experience of fear, anger, and concern, which Sandman has termed “outrage”. The greater the outrage, the greater the perceived risk, even if the real danger is low [20,21]. In this context, proper communication should bridge the gap between experts on risk management and the population, contributing, at the same time, to bringing the risk perception back to the real level [16,20,21]. 

During the last decades, Sandman’s theory has been largely applied in risk communication controversies of different natures, mostly referring to public health concerns (e.g., vaccines, pandemics, etc.). Indeed, Sandman’s model of risk, hazard, and outrage provide a useful heuristic tool to understand message design and effectiveness; by contrast, the model lacks an accurate measurement method [22].

In 1998, Sandman’s approach to outrage management led to the development of “Outrage Prediction & Management” (OPM) software (EMSoftCorp, Portland, OR, USA). The software was designed to allow users to evaluate the stakeholders’ outrage level, and to decide how to manage it, simulating the answers an expert (i.e., Sandman) would have formulated. 

Since Sandman’s theory also has implications in economics, the software was initially designed to increase the productivity of business companies. To date, it is possible to access a free version of OPM, which could be useful to better understand and predict the level of public outrage also related to various public health issues, such as the drinking water supply. 

Based on these premises, the present study aims at understanding the effectiveness of the risk communication strategies related to water intended for human consumption, in order to evaluate how much the community outrage and population distrust are attributable to faults in communication. Our analysis is based on the application of Peter Sandman OPM. To our best knowledge, this is the first time that OPM has been applied in public health threats.

## 2. Materials and Methods 

### 2.1. Study Setting

Sardinia is the Italian Region with the highest population distrust of drinking water quality [11,15,23]. Several studies have shown the critical issues related to the island’s water resources and subsequent drinking water production and distribution [11,24,25,26,27,28,29]. 

The region has been suffering from a longstanding and recurrent drought leading to a widespread qualitative decline of water supplies. To tackle the situation, various projects aimed at regulating the course of water streams have been implemented [30], and the region has the highest number in Italy of artificial basins intended for drinking water production. The basins represent the main water source for most of the Sardinian population, even though they are notoriously exposed to anthropic contamination and provide water with a poor quality. As a result, the purification systems often fail, and the production of drinking water represents a potential health risk [11,29].

This condition has led, on the one hand, to an increase in the ordinances issued during the last years by Sardinian Municipalities and on the other, to a great population unease, which is reflected in the largest consumption of bottled water throughout the national territory [11,16,23,31]. 

Poor quality, declining organoleptic properties, and frequent supply interruptions have constantly repeated through the years, affecting the population’s daily life [32]. Consequently, the Sardinian population is highly suspicious of the quality of public drinking water and discourages its consumption. Moreover, its insularity, alongside the reduced availability of alternative water supply sources, make it an optimal studying context for outrage assessment [24]. 

### 2.2. Collection and Categorization of Ordinances

According to Italian laws [8], the drinking water quality is verified by the Local Health Trusts, with variable frequency depending on the volumes of water distributed and the population supplied. If water quality does not comply with the legislative standards, the Health Authority issues specific ordinances, with the following objectives: (i) to promptly advise the population about the related health risks due to an unsafe water consumption; (ii) to prescribe the interruption of supply or limit of water consumption for domestic use (such as bathing and showering), rather than for use in food preparation.

Urgent ordinances, published in the District Bulletin of the Municipality, are also communicated through various mass media (such as their institutional websites). Thus, we collected the ordinances available online, issued during the years 2010–2015 in Sardinia, referring to data published in a previous study [11].

Secondly, two investigators independently read the ordinances, in order to evaluate their contents and to categorize them based on the completeness of information reported (i.e., causes of the abnormality detected; unacceptable parameters; concentration values; possible health risks; food use limitation; domestic use allowed).

Reading the ordinances allowed the Authors to better understand how the Authorities managed the risk information and how they relayed this information to the population, acquiring the necessary knowledge to objectively apply OPM and to obtain the prediction of the stakeholders’ outrage level.

### 2.3. OUTRAGE Prediction & Management: Software Application

In the 1990s, an Australian consulting firm (called QEST Consulting Engineers, Melbourne, Australia) began to build an expert systems software based on Peter Sandman’s outrage theory.

In 1998, “OUTRAGE Prediction & Management” (EMSoftCorp, Portland, OR, USA) was released into the market. In 2007 new software, called “Engage”, was released and replaced OPM, which was no longer updated. Therefore, OPM is now a freeware which can be downloaded from Sandman’s website [33]. In fact, it may be installed under a compatible mode on new-generation personal computers. 

Building the software required Sandman to formulate a large number of questions, with the aim of enabling the software to interpret any controversy, simulating what a communication expert would have asked to evaluate the level of outrage generated by the dispute. Overall, Sandman formulated 227 questions, which were included in the software.

The next step was to draft answers to the aforementioned questions. The answers that Sandman proposed included not only correct but also incorrect answers, in order to capture the misjudgments of the situation analyzed. 

Overall, the software is a collection of questions and the suggested answers that a user can choose from a drop-down menu. The resulting assessment led to the outrage prediction, based on values weighted and validated by Sandman. Thus, installing the software allowed us to assess the community outrage level in an assisted mode, guiding us during the analysis and the implementation in this peculiar public health context.

The first step in the assessment process consists in creating a “Situation Work”, linked to a “Situation Definition”, a “Stakeholder Listing and Analysis” and, finally, the “Outrage Assessment”.

**Figure 1:**
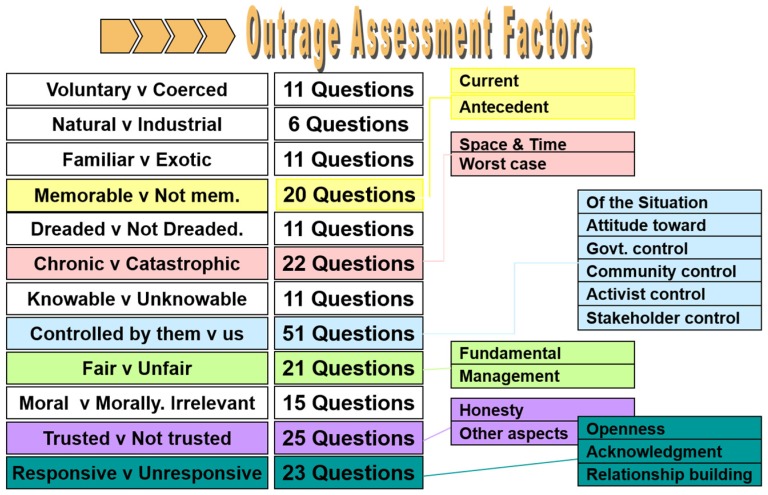
The 12 principal outrage components according to Peter Sandman’s Outrage theory (Available on http://www.psandman.com/handouts/sand58.pdf), and the questions formulated by Sandman in relation to each component. The resulting questions were used to build OPM.

The “Outrage Assessment” can be performed both for all of the important stakeholders, and for a single stakeholder category, and requires users to respond to 227 questions within the 12 components (risk communication variables) of Peter Sandman’s theory [34] (Figure 1).

According to Peter Sandman and the software creators, the questions have different weights in scoring an outrage factor, and the outrage factors in making up a total “outrage score.” As a result, firstly, a mathematical model was used to weight both the questions and factors; secondly, as the results obtained by Sandman after testing the software were not completely satisfactory, an algorithm that reliably replicated Sandman’s intuitive outrage assessment was finally set up. Thus, OPM was validated by Peter Sandman.

Overall, the outrage prediction score is based on a weighted total of all the outrage scores for each component, normalized in order to set the maximum possible outrage score at 1000. In particular, a final score of between 0 and 400 represents a low outrage prediction; from 400 to 600 the outrage is considered as acceptable; over 600 the outrage is unacceptable.

Once the total outrage score is acquired, the researcher can review its main contributors and work on outrage reduction strategies. 

Accordingly, the software application consisted of creating the “Situation Work” referring to the quality of the water intended for human consumption in Sardinia. As a result, based on the contents of 417 ordinances, the Authors created the Situation Work and answered the questions asked by the software. The inconsistencies were solved by consensus of all the Authors.

## 3. Results

**Figure 2:**
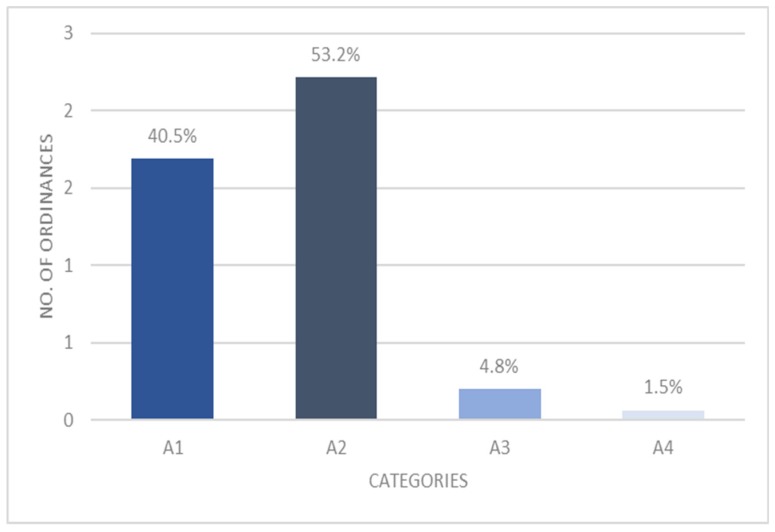
Number of ordinances and their relative frequencies within the categories of information. A1: ordinances with no information available about the drinking water problems detected; A2: ordinances reporting the unacceptable parameter(s); A3: ordinances reporting abnormal parameter(s) and concentration value(s); A4: ordinances reporting all the information.

During the years 2010–2015, 417 ordinances were published on the institutional websites of Sardinian Municipalities. The two investigators’ independent reading allowed for their ascription to 4 categories with an excellent reliability (Kappa (95%CI) = 0.992 (0.98–1.0)), starting from the most incomplete (A1), to the richest in information (A4) (Figure 2). Of these, 169 ordinances (40.5%) did not specify any information but a generic “unsuitable water” warning. Thus, they were ascribed to category A1. The majority (53.2%) of the ordinances analyzed (222/417) specified only the unacceptable parameters and were ascribed to the A2 category. The ordinances reporting abnormal parameters and concentration values were 20/417 (4.8%), whereas only 6/417 (1.5%) showed completeness of information given.

### 3.1. Outrage Assessment

**Figure 3:**
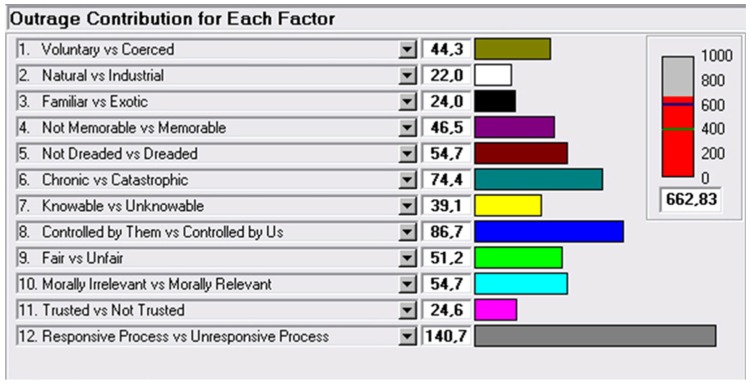
The contribution of each factor on the outrage assessment score obtained.

The results obtained during the outrage assessment software application are shown in Figure 3. 

The final score of 662 is the sum of each contributor, which was higher than the threshold (600) that represents the marginal risk, and the red bar indicates that the population perceives the risk as unacceptable. The bar graph shows the score for each factor.

### 3.2. Outrage Management

Based on the identified critical factors, the software allowed us to readdress some of the same outrage assessment questions, concentrating on the highest-scoring factors, with the aim of implementing new strategies to correct the shortcomings in communication. In practice, by understanding reputation problems the user could then try to manage them.

OPM automatically identified the more advantageous factors to change. Changing answers means contextually changing strategies, bringing the outrage score down.

**Figure 4:**
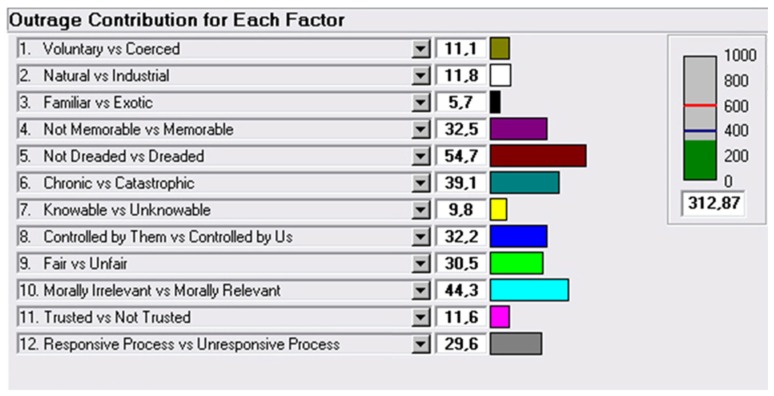
The contribution of each factor to the outrage management score obtained.

Figure 4 shows the results of the Management Options Analysis, which allowed the achievement of a score of 312 in the outrage assessment.

## 4. Discussion

The analysis of the ordinances’ structure and contents showed how often information about health risks due to drinking water abnormalities was omitted. From the present study, it emerged that 53.2% (category A2) of the ordinances issued reported only the unacceptable parameter. This format represents a kind of standard communication used by the Health Authority.

The situation becomes even more serious if we consider that 40.5% of the ordinances observed did not report any information about the abnormalities occurring. As a result, the Sardinian population does not receive sufficiently clear information from the Authorities.

The application of the OPM software allowed for an evaluation of the community outrage level, even though it was created in order to predict and manage the outrage for businesses. Accordingly, our study demonstrated the flexibility of the tool, and the results obtained are consistent with those that emerged in a previous study [24], where an outrage indicator showed that drinking water supply interruption or limitation is an outrageous threat in Sardinia.

Taking a closer look at the outrage assessment performed, factors that mainly contributed to the final score were: “Controlled by them/by us”; “Responsive/Unresponsive process”; and “Chronic/Catastrophic”. These factors indicate that the population demands more direct participation in the process, as well as clear information about the real risks related to drinking water consumption. Moreover, the situation perceived will deteriorate in the future.

The subsequent outrage management application gave noteworthy results. During the new strategy simulation, the most outrageous factor (“Responsive/Unresponsive process”) disappeared among the critical factors, suggesting that there should be more collaboration between categories of stakeholders. In particular, changing the answers within the two most critical factors (“Controlled by them/by us”; “Responsive/Unresponsive process”), which refer to the relationship between the population and the public administration, the outrage assessment score decreased to 510.02, below the critical threshold of 600 (acceptable outrage level). 

This means that communication strategies should be particularly focused on restoring the reputation of the Authorities and the perception that the public has in its regard. As a result, the continuation of the study foresees the drafting of guidelines for correct management of the risk communication, based on consolidated knowledge and the best practices actually available for a proper risk communication. For example, as suggested by Venette and approved by the International Union of Food Science and Technology Scientific Council [35], listening to public concerns are key in providing clear messages and properly understanding how effective information exchange can reduce harm during catastrophic events. Thus, owing to the low attention paid to proper risk communication through the ordinances issued during the last years in Sardinia, it is pivotal to re-consider the contents and how the information is given to the community (clarity, comprehensiveness, completeness, etc.). 

Before drawing conclusions, we would like to underline the possible weaknesses or strengths of our findings. 

On the one hand, the software requires users to answer the 227 questions, and several hours are needed to complete the assessment; on the other hand, the assisted mode reports several examples and explanations which are useful to correctly answer all the questions, limiting possible misunderstandings. 

Moreover, the outrage assessment performed by OPM aims at evaluating the community outrage based on the real situation of a specific controversy, and it is far from a “customer satisfaction” analysis. Thus, the software application requires exact knowledge of the situation described. As such, reading the ordinances allowed us to construct a robust background of knowledge to define the Situation Work and objectively answer the questions, giving a real picture of the population concerns due to the risk communication strategies related to unsafe drinking water consumption, which was implemented by the Sardinian Authorities during the last years.

Furthermore, the high level of outrage we found had already been highlighted by national reports [15,32], but only recently has it been confirmed through the application of the Sandman’s theory [24].

Overall, the OPM software application resulted in an accurate description of the highest distrust of the drinking water safety in Sardinia, and the results are consistent with those nationally reported. Even though the software is out-of-date (it needs to be run under a compatible mode, or to use an old operating system on a personal computer), it is useful and freely available to the users, and it appears to be a flexible tool to assess even public health controversies.

## 5. Conclusions

Water intended for human consumption is key in ensuring healthy and salutogenic cities [36,37]. Despite the importance of the issue [1,2], industrialized countries and urban areas are still facing difficulties to distribute potable water to the population. Italy is not exempt from qualitative and quantitative abnormalities [8], and Sardinia represents one of the Italian regions with the highest population distrust of drinking water safety.

Ordinances issued by the Health Authority represent the main risk communication tool according to Italian law. The absence of a specific regulation regarding mandatory contents to report made the majority of the ordinances issued and published unable to appropriately communicate the health risks arising from unsafe drinking water consumption.

Thus, on the one hand, ordinances should be more comprehensive and complete with information and on the other, it could be useful to adopt other strategies in supporting ordinances (e.g., updated informative drinking water sections published on institutional websites; drinking water labels that report the characteristics of potable water, in order to inform the population every time that routine analyses are made, etc.). Accordingly, the perspectives of the present study acknowledge the drafting of guidelines, which should be focused on re-considering how to give the information to the citizens, in order to improve the comprehensiveness and completeness of the contents of the ordinances, ensuring that consumers are aware of the risks associated with the tap water, and thereby use it safely.

In this context, OPM is a useful, flexible tool, able to predict the community outrage and to suggest actions which should be implemented for its management.
